# The triglyceride glucose index is associated with future cardiovascular disease nonlinearly in middle-aged and elderly Chinese adults

**DOI:** 10.1186/s12902-022-01157-6

**Published:** 2022-10-03

**Authors:** Zixiang Ye, Enmin Xie, Yanxiang Gao, Peizhao Li, Yimin Tu, Ziyu Guo, Qing Li, Yaxin Wu, Xiaozhai Yu, Yike Li, Changan Yu, Jingang Zheng

**Affiliations:** 1 Department of Cardiology, Peking University China-Japan Friendship School of Clinical Medicine, Beijing, 100029 China; 2 Graduate School of Peking Union Medical College, Chinese Academy of Medical Sciences and Peking Union Medical College, Beijing, 100029 China; 3 Department of Cardiology, China-Japan Friendship Hospital, Beijing, 100029 China

**Keywords:** Triglyceride glucose index, Cardiovascular disease, Adults, Chinese, Insulin resistance

## Abstract

**Objective:**

We aimed to investigate the association between triglyceride glucose index and cardiovascular disease (CVD) development in the Chinese middle-aged and elderly population using the China Health and Retirement Longitudinal Study dataset 2011–2018.

**Methods:**

Basic characteristics of participants, including sociodemographic information, and health conditions, were acquired. Logistic regression analyses and restricted cubic spline regression analyses were conducted to investigate the association between the triglyceride glucose index and future CVD risks. Subgroup analyses were performed to evaluate potential interaction.

**Results:**

Seven hundred fifty-three of 6114 (12.3%) participants have developed CVD in 2018 over an approximately 7-year follow-up. The logistic regression analysis exhibited that compared to the lowest triglyceride glucose index group, the multivariable OR for future CVD was 0.985 (95%CI 0.811–1.198) in the T2 triglyceride glucose index group and 1.288 (95%CI 1.068–1.555) in the T3 TyG index (P for trend 0.006). The restricted cubic spline regression analysis showed the nonlinear association between triglyceride glucose index and CVD incidence; the cut-off values were 8.07 and 8.57, respectively, after total adjustment. Gender, fast blood glucose, and triglycerides interacted with triglyceride glucose index and CVD except for BMI.

**Conclusion:**

The triglyceride glucose index was nonlinearly related to the risk of future cardiovascular disease in the middle-aged and elderly Chinese population.

## Introduction

The incidence of cardiovascular disease is related to a variety of influencing factors, such as diabetes, and chronic kidney disease, which has added an enormous burden to the national health finance [1]. Therefore, it is crucial to identify the population susceptible to cardiovascular disease for early intervention to reduce cardiovascular disease incidence and comorbidities.

Insulin resistance (IR) refers to the decrease in the efficiency of insulin to promote glucose uptake and utilization and the compensatory secretion of excessive insulin by the body to produce hyperinsulinemia to maintain the stability of blood sugar, serving as one of the main physiological pathways of type 2 diabetes and metabolic syndrome [2]. Recently, it has been reported that there was a close relationship between insulin resistance and cardiovascular disease leading to the occurrence of cardiovascular disease due to the low-grade inflammation increased by overproduction of reactive oxygen species and advanced glycation end products [3]. At present, traditional methods of measuring insulin resistance, like the homeostasis model assessment of IR, are cumbersome and labor-intensive with limited clinical value. Therefore, there is an urgent need for a new indicator to measure insulin resistance conveniently and effectively.

The triglyceride glucose (TyG) index is a combined triglyceride and fasting blood glucose (FBG) index. Recent studies have shown that the TyG index is a reliable surrogate marker for insulin resistance, with high sensitivity and specificity [4]. Many researches have reported that the TyG index is associated with high-risk factors of cardiovascular disease such as diabetes [5], metabolic syndrome [6], and hypertension [7]. Some studies have shown that the TyG index is related to coronary artery calcification scores [8] and arterial stiffness [9]. However, the prospective cohort studies on whether the TyG index is associated with new-onset cardiovascular disease are still lacking [10].

The current study explores the relationship between the TyG index and the risk of developing cardiovascular disease in the Chinese middle-aged and elderly population in a large-scale prospective cohort study from the China health and retirement longitudinal study (CHARLS). This study is a follow-up of our previous study from the same cohort, which demonstrated that the elevated TyG index strengthens the association between diabetes and cardiovascular disease [11].

## Methods

### Study design and population

The dataset CHARLS is a nationwide prospective cohort study enrolling adults aged ≥45 from 28 provinces in China between June 2011 and March 2012. A total of 17,708 participants at baseline completed the assessment via a standardized questionnaire in the face-to-face interview [12]. The measurement of basic vital signs, including blood pressure, height, and weight were performed, and blood samples were acquired. The baseline response rate was 80.5%. The Peking University institutional review board approved the CHARLS study (IRB00001052–11015), and informed consent from all participants enrolled in this study was obtained. All methods were performed in accordance with the relevant guidelines and regulations.

**Fig. 1:**
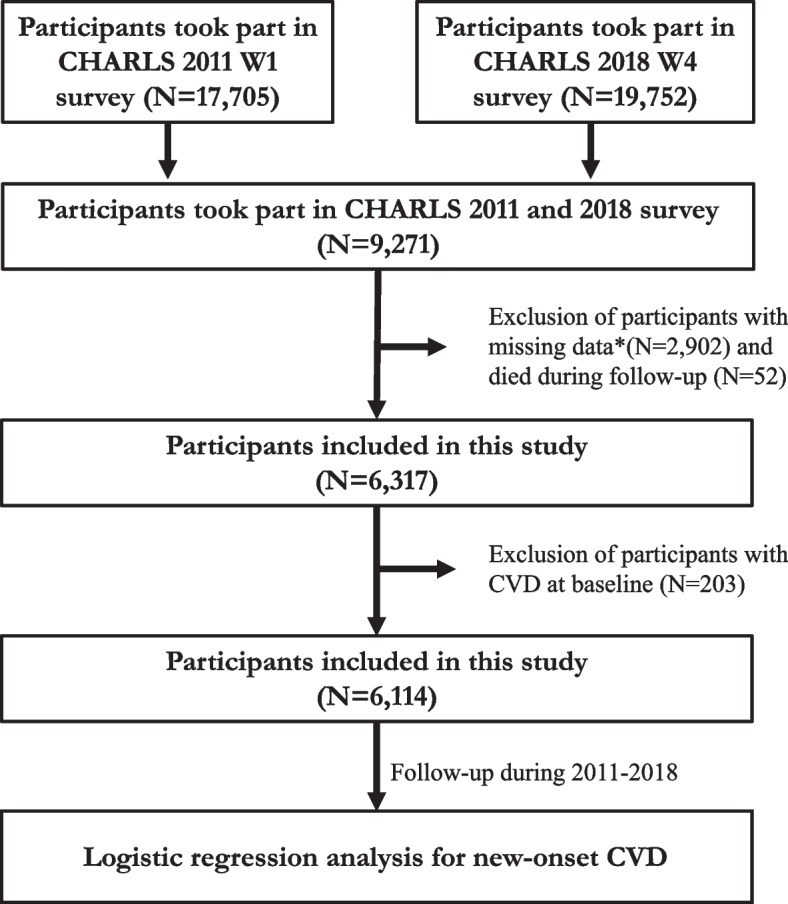
Flowchart of the procedure extracting the patients from CHARLS. *Missing data includes age, sex, FBG, TG, CVD, creatinine condition

This article extracted the baseline data in 2011 (Wave 1) and follow-up data in 2018 (Wave 4). All the participants in 2011 were followed up every 2 years and invited to participate in the Wave 4 follow-up survey in 2018 (Fig. 1).

### Data collection and definition

Professional researchers collected sociodemographic information via interviewing participants, including age, gender, smoking condition (smoker or nonsmoker), alcohol drinking condition (drinker or nondrinker), education level (low grade, medium grade, and high grade), health conditions including hypertension, diabetes, CVD, dyslipidemia, and medication usage including antihypertensive drugs, hypoglycemic drugs, and lipid-lowering drugs. The HEM-7200 electronic monitor was performed to measure static blood pressure three times. Fasting blood samples were collected and transported to Beijing at − 80 °C at the Chinese Center for Disease Control and Prevention. The laboratory test of biomarkers, including fasting blood glucose (FBG), glycated hemoglobin, total cholesterol (TC), high-density lipoprotein cholesterol (HDL-C), low-density lipoprotein cholesterol (LDL-C), triglycerides (TG), and hemoglobin were conducted at the Clinical Laboratory of Capital Medical University.

The definition of hypertension was systolic blood pressure ≥ 140 mmHg or diastolic blood pressure ≥ 90 mmHg, or professional doctor-diagnosed hypertension or under antihypertensive medication. Diabetes was defined as fast blood glucose (FBG) of more than 125 mg/dl or Hemoglobin A1c of more than 6.5%; the doctor diagnosed diabetes or under antidiabetic medications in the past 2 weeks. Chronic kidney disease (CKD) was defined as eGFR < 60 mL/min/1.73m^2^ [13].

TyG index was evaluated based on the TG and FBG concentration according to the equation: ln [TG (mg/dl) × FBG (mg/dl)/2]. The Incident of developing CVD events was defined as the positive answer to the question of whether had doctor-diagnosed heart disease (including heart attack, coronary heart disease, angina, congestive heart failure, or other heart problems) or stroke during the follow-up period, which was regarded as the outcome of this study.

### Statistical analysis

Normally distributed continuous variables were described as means and standard deviation (SD), while nonnormally distributed continuous variables were presented as medians and interquartile ranges. Categorical variables were described as the frequency with percentage. Baseline characteristics were summarized according to the tertiles of TyG index (T1, T2, T3) and the incidence of CVD using the χ2 test, One-Way ANOVA, or Mann-Whitney U test, as appropriate. Univariable and multivariable logistic regression analyses were performed to estimate the relationship between the TyG index and incidence of future CVD fitting three logistic models. Model1, without any adjustment only included the TyG index. Age and gender were adjusted in Model2. Model3 was a fully adjusted model with adjustment for age, gender, BMI, education level, TC, LDL-C, HDL-C, uric acid, BUN, history of hypertension, dyslipidemia, and CKD, smoking and drinking. In addition, the multivariate restricted cubic spline (RCS) regression using 3-knotted at 25th, 50th, and 75th was performed to evaluate the shape of the potential nonlinear associations between the TyG index and new-onset CVD risk. Subgroup analyses were conducted by age, gender, BMI, the value of FBG and TG, and P for interaction were evaluated respectively. When *P* < 0.05, it was regarded as statistically significant. All statistical analyses were conducted in R statistical software (4.0.6, Vienna, Austria).

## Results

### Baseline characteristics

The baseline features of participants enrolled from CHARLS according to the trisection of the TyG index were described in the previous study in detail. The average TyG index was 8.66 ± 0.65 (T1 8.01 ± 0.25, T2 8.58 ± 0.15, T3 9.38 ± 0.49). Six thousand one hundred fourteen participants (2776 men and 3338 women) were enrolled in this study in total with a mean age of 57.87 ± 8.98. The content of BUN, glucose, creatinine, TC, TG, HDL-C, LDL-C, uric acid, and eGFR, as well as the prevalence of hypertension and CKD, were significantly different among various TyG index groups. The comparison of the group that developed CVD in 2018 and non-CVD groups was illustrated in Table 1. The population who developed CVD had a higher TyG index, BMI, glucose, TG, and uric acid and was more likely to suffer from hypertension, dyslipidemia, and asthma.

**Table 1:** The baseline characteristics of participants classified by CVD

	Total	Non-CVD	CVD	***P value***
**N**	6114	5361	753	
**TyG index**	8.66 (0.65)	8.65 (0.64)	8.71 (0.72)	0.023
**TyG (%)**				
T1	2038 (33.3)	1799 (33.6)	239 (31.7)	0.028
T2	2038 (33.3)	1807 (33.7)	231 (30.7)	
T3	2038 (33.3)	1755 (32.7)	283 (37.6)	
**Education (%)**				0.202
Low	2876 (47.0)	2521(47)	355(47.1)	
Median	3081 (50.4)	2706(50.5)	375(49.8)	
High	157 (2.6)	134(2.5)	23(3.0)	
**AGE(y)**	57.87 (8.98)	57.94 (9.05)	57.41 (8.51)	0.133
**Male (%)**	2776 (45.4)	2399 (44.7)	377 (50.1)	0.007
**BMI**	23.67 (4.98)	23.60 (4.92)	24.17 (5.38)	0.003
**BUN (mg/dl)**	15.66 (4.37)	15.67 (4.40)	15.63 (4.21)	0.81
**Glucose (mg/dl)**	108.72 (33.12)	108.29 (31.91)	111.77 (40.66)	0.007
**Creatinine (mg/dl)**	0.77 (0.18)	0.77 (0.18)	0.78 (0.18)	0.147
**TC (mg/dl)**	193.22 (37.80)	193.30 (37.80)	192.64 (37.82)	0.658
**TG (mg/dl)**	129.55 (95.12)	128.65 (93.86)	136.00 (103.52)	0.047
**HDL-C (mg/dl)**	51.61 (15.25)	51.70 (15.21)	51.00 (15.57)	0.241
**LDL-C (mg/dl)**	116.17 (34.35)	116.35 (34.24)	114.90 (35.11)	0.279
**CRP (mg/l)**	2.44 (6.67)	2.43 (6.80)	2.47 (5.68)	0.893
**Uric Acid (mg/dl)**	4.39 (1.21)	4.37 (1.21)	4.49 (1.25)	0.016
**Hypertension (%)**	1125 (18.4)	894 (16.7)	231 (30.7)	< 0.001
**Dyslipidemia (%)**	393 (6.4)	305 (5.7)	88 (11.7)	< 0.001
**Asthma (%)**	172 (2.8)	139 (2.6)	33 (4.4)	0.008
**Smoking (%)**	2324 (38.0)	2044 (38.1)	280 (37.2)	0.646
**Drinking (%)**	1927 (31.5)	1703 (31.8)	224 (29.7)	0.283

### The odds ratio of the TyG index on the incidence of future CVD

**Fig. 2:**
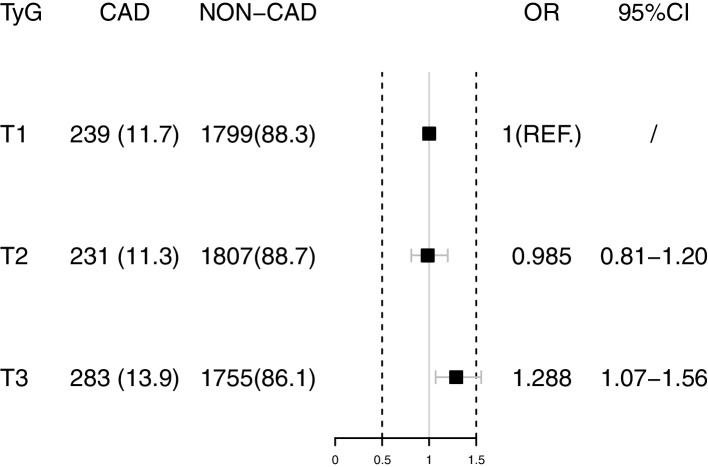
The logistic regression analysis showed the multivariable OR for CVD decreased in the T2 group and increased in the T3 group compared with the T1 group

**Fig. 3:**
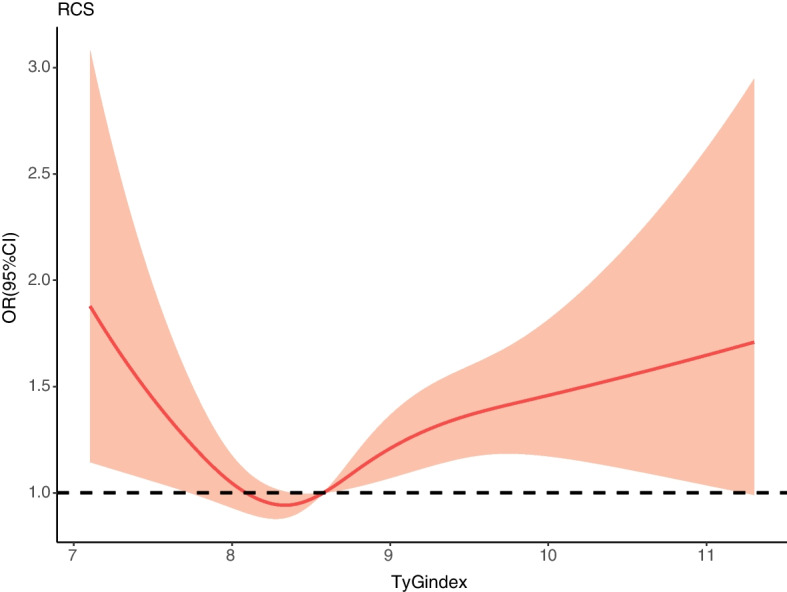
The multivariable restricted cubic spline regression result exhibited the J-shape relationship between TyG index and the new-onset CVD incidence after adjusted for age, sex, BMI, education level, TC, LDL-C, HDL-C, uric acid, BUN, history of hypertension, dyslipidemia and CKD, smoking, drinking. The cut-off values were 8.07 and 8.57 respectively

Seven hundred fifty-three of 6114 (12.3%) participants developed CVD in 2018 after the about 7 years follow-up period. After complete adjustments of age, gender, education, BMI, history of hypertension, dyslipidemia, asthma, and CKD, logistic regression analysis exhibited the multivariable OR (Model 3, Fig. 2) for new-onset CVD decreased in the T2 TyG index group (OR 0.985 95%CI 0.811–1.198) and increased in the T3 TyG index (OR 1.288 95%CI 1.068–1.555) compared with the lowest TyG index tertiles (Table 2, P for trend 0.006). The multivariable restricted cubic spline regression analysis exhibited that the nonlinear relationship between the TyG index and the new-onset CVD incidence was J-shape, the cut-off values were 8.07 and 8.57, respectively, after total adjustment (Fig. 3).

**Table 2:** The association between various TyG index groups and CVD

TyG index	Model1	Model2	Model3
**T1**	ref.	ref.	ref.
**T2**	0.962(0.794–1.166)	0.978(0.807–1.186)	0.985(0.811–1.198)
**T3**	1.214(1.009–1.459)	1.243(1.033–1.495)	1.288(1.068–1.555)
**P for trend**	0.029	0.018	0.006
Model2	adjusted for age, gender		
Model3	adjusted for age, gender, BMI, education, smoking, drinking, LDL-C, HDL-C, TC, uric acid, BUN, history of hypertension, dyslipidemia, CKD

### Subgroup analysis

**Fig. 4:**
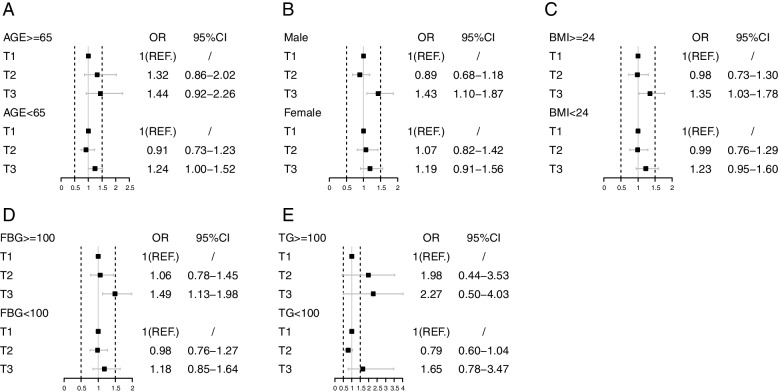
The subgroup analysis for association between TyG index and CV classified by age (**A**), gender (**B**), BMI (**C**), FBG (**D**) and TG (**E**)

Subgroups analyses were performed to analyze the relationship between the TyG index and future CVD risk stratifying by age, gender, BMI, and the value of FBG and TG at baseline. Gender, FBG and TG were shown to have an interaction with the TyG index and future CVD. The J-shape relationship between the TyG index and future CVD risk is still found in younger participants, men, both overweight and normal participants, and population without increased FBG or TG levels. There was a positive correlation between the TyG index and future CVD risk in older participants, women, and participants with increased FBG or TG levels (Table 3, Fig. 4).

**Table 3:** The subgroup analysis result of multivariable-adjusted OR for association between TyG index and CVD

	Case	Total	T1	T2	T3	P for trend	P for interaction
**Age**							
**≥ 65**	147	1355	ref.	1.318(0.858–2.023)	1.444(0.924–2.257)	0.045	0.165
**<65**	605	4152	ref.	0.909(0.729–1.132)	1.235(1.004–1.521)	0.013	
**Gender**							
**Male**	377	2776	ref.	0.898(0.681–1.184)	1.431(1.097–1.867)	0.002	0.003
**Female**	376	3338	ref.	1.079(0.816–1.426)	1.192(0.910–1.562)	0.033	
**BMI**							
**≥ 24**	355	2687	ref.	0.977(0.732–1.304)	1.352(1.027–1.782)	0.032	0.570
**<24**	398	3427	ref.	0.988(0.757–1.288)	1.230(0.951–1.592)	0.168	
**FBG, mg/dl**							
**≥ 100**	419	3458	ref.	1.062(0.780–1.447)	1.499(1.131–1.989)	0.002	0.020
**<100**	334	2656	ref.	0.982(0.757–1.276)	1.182(0.850–1.642)	0.541	
**TG, mg/dl**							
**≥ 100**	429	3234	ref.	1.983(0.440–3.526)	2.267(0.505–4.029)	0.291	0.017
**<100**	323	2880	ref.	0.795(0.604–1.046)	1.645(0.781–3.466)	0.088

## Discussion

Based on the nationally representative and prospective cohort CHARLS, our previous study demonstrated that the TyG index, which served as the surrogate of insulin resistance, plays a crucial role in exacerbating the association between DM and CVD risk. In this study, we further explored the relationship between the TyG index and the risk of future CVD incidence in nonlinear fashion in the Chinese adults’ group. In the high TyG index groups (TyG index>8.57), the value of the TyG index was positively correlated to the increased incidence of future CVD after total adjustment. This finding was in accordance with subgroup analyses in younger participants, men, overweight and normal participants, and a population without increased FBG or TG levels.

The TyG index is a precise and straightforward biomarker of insulin resistance calculated utilizing the TG and FBG concentrations [14]. Insulin resistance was regarded as a risk factor for hypertension, diabetes, and metabolic syndrome leading to imbalanced glucose metabolism with oxidative stress and inflammation as well as endothelial dysfunction [15]. Endothelial dysfunction was closely related to CVD development, including atherosclerosis and acute coronary syndrome [16]. Thus, IR was significantly correlated to CVD development [17], and a high TyG index level, as an alternative biomarker for IR, could result in adverse vascular remodeling via oxidation and systemic inflammation and produce lipotoxicity in the cardiovascular system [18]. This provides strong evidence that a high TyG index might be a possible predictable biomarker for developing CVD. Furthermore, according to a previous clinical study, the TyG index is a significant predictor of coronary artery calcification development [19] and was a more vital predictive factor of CVD than hemoglobin A1c in diabetic patients [20].

Our research was in line with previous studies that the association between the value of the TyG index and the CVD risk was not linear. A recent dose-response meta-analysis illustrated that a high TyG index was related to major adverse cardiovascular events in the acute coronary syndrome population in a non-linear fashion, with statistical significance reaching approximately TyG index 8.9 and increased non-linearly [21]. In a large white European cohort (Vascular Metabolic CUN cohort), the highest level TyG index (TyG index>8.81) population had a 2.32-fold higher risk of having CVD than the lowest TyG index group(6.40<TyG index<7.87). There was an L-shaped association between the level of TyG index and incidence of CVD, from which the risk surged approximately 8.45 [22]. Another large-scale cohort study also reported the nonlinear dose-dependent relationship between increased TyG index and high risk of future CVD in 6078 participants. Ranging from 8.50 to 10.75, an increasingly higher TyG index value was correlated to a higher CVD risk [23]. The current study showed that a higher TyG index (TyG index>8.57) was related to an elevated risk of developing CVD in the middle-aged and elderly Chinese population.

There was currently insufficient evidence to suggest how gender differences regulate the effects of the TyG index on cardiovascular risk [24]. In current subgroup analyses, the nonlinear association was prominent in man. As for age, in the Tehran Lipid and Glucose Study, which enrolled 7521 Iranians, the association between the TyG index and CVD risk was more significantly conspicuous among the younger population, which was in accordance with our subgroup analysis results [25]. TyG index was also related to diabetes because IR is regarded as the crucial pathogenesis factor in developing diabetes. BMI is one of the recognized risk factors for the development of diabetes and CVD [26]. Some studies reported that the TyG index partially mediated the relationship between BMI and the development of CVD and DM. Being overweight leads to an increase in free fatty acids, which triggers IR. It further inhibits insulin signal transduction and increases glucose transport in the liver [27, 28]. Besides, obesity-related inflammatory factors promote lipolysis and triglyceride synthesis in the liver and further promote fatty acid esterification to aggravate the occurrence of hyperlipidemia [17]. TyG index, calculated utilizing the TG and FBG concentrations, is a reasonable mediator between BMI and the risk of developing future CVD, partially explaining the J shape relationship between TyG index and future CVD incidence [29].

The strength of the current study is that the longitudinal national representative cohort sample was performed with rigorous procedure and protocol for baseline information collection and follow-up. However, some limitations in this study still should be considered. First, all participants were only middle-aged and elderly Chinese adults without representing people of from all ages and various ethnic populations. Second, we could not differentiate various lipid-lowering drugs such as statins and fibrates or different anti-diabetic and anti-hypertension medications based on CHARLS. The impact of other medical treatments should be adequately controlled in the prospective cohort studies, considering that various medications might impact future CVD incidence. Last, this study could not fully explain the potential pathogenic mechanisms. Further research should be warranted.

To sum up, the TyG index was correlated with the incidence of developing CVD with the nonlinear relationship. TyG index was positively associated with risks of CVD in a high TyG index value population. In addition, a high TyG index can be a significant and potential predictor of future CVD incidence.

## Data Availability

The data that support the findings of this study are available from the China Health and Retirement Longitudinal Study (CHARLS) but restrictions apply to the availability of these data, which were used under license for the current study, and so are not publicly available. Data are however available from the author Zixiang Ye (yezxiang@yeah.net) upon reasonable request and with permission of CHARLS.

## Abbreviations

95%CI: 95% confidence intervals
CVD: Cardiovascular disease
IR: Insulin resistance
TyG: Triglyceride glucose
MACE: Major adverse cardiovascular events
CHARLS: China Health and Retirement Longitudinal Study
TG: Triglycerides
FBG: Fasting blood glucose
TC: Total cholesterol
hsCRP: High-sensitivity C-reactive protein
HDL-C: High-density lipoprotein cholesterol
LDL-C: Low-density lipoprotein cholesterol
BUN: Blood urea nitrogen
DM: Diabetes mellitus
eGFR: Estimated glomerular filtration
CKD: Chronic kidney disease
IQR: Interquartile ranges
SD: Standard deviation
